# Phosphate solubilizing fungi enhance insoluble phosphate dissolution via organic acid production: mechanisms and applications

**DOI:** 10.3389/fmicb.2025.1600231

**Published:** 2025-05-16

**Authors:** Ying Ma, Shenghao Chen, Shuang Liu, Linjia Guo, Chaochun Zhang, Xinxin Ye, Da Tian

**Affiliations:** ^1^Anhui Province Key Lab of Farmland Ecological Conservation and Nutrient Utilization, College of Resources and Environment, Anhui Agricultural University, Hefei, China; ^2^Anhui Province Engineering and Technology Research Center of Intelligent Manufacture and Efficient Utilization of Green Phosphorus Fertilizer, College of Resources and Environment, Anhui Agricultural University, Hefei, China; ^3^Key Laboratory of JiangHuai Arable Land Resources Protection and Eco-Restoration, Ministry of Natural Resources, College of Resources and Environment, Anhui Agricultural University, Hefei, China

**Keywords:** phosphate solubilizing fungi, insoluble phosphates dissolution, organic acid, phosphate biofertilizer, phosphorus release

## 1 Introduction

Phosphorus (P) is an essential element for plant growth, which functions in photosynthesis, root development, and nucleotide incorporation (Bisson et al., 2017). However, only 0.1–0.5% of the total soil P is available for plants to absorb (Sharma et al., 2013). Generally, most P in the soil is insoluble and adsorbed, significantly affecting plant accessibility and crop yield (Tian et al., 2020). The commonly insoluble phosphates (IPs) in soils usually include ferric phosphate (FePO_4_, Fe-P), aluminum phosphate (AlPO_4_, Al-P), and tricalcium phosphate (Ca_3_(PO_4_)_2_, Ca-P) (Tian et al., 2024). These IPs are distributed across various soil types, which limits crop yields and phosphorus use efficiency (Tian et al., 2021a).

The input of chemical phosphate fertilizer can promote plant adsorb P and increase crop yield. However, over 60% of P fertilizers rapidly react with soil metal cations (e.g., Ca^2+^, Fe^3+^) and become immobilized as IPs (Jayashree et al., 2011; Mahdi et al., 2020). According to the statistics, the IPs stored in soils could alleviate the expected P shortages over the next 50 years (Zhu et al., 2018). Therefore, enhancing the development and utilization of this stored P in agricultural soils is crucial for sustainable P management in the future (Tian et al., 2021b, 2024).

**Figure 1:**
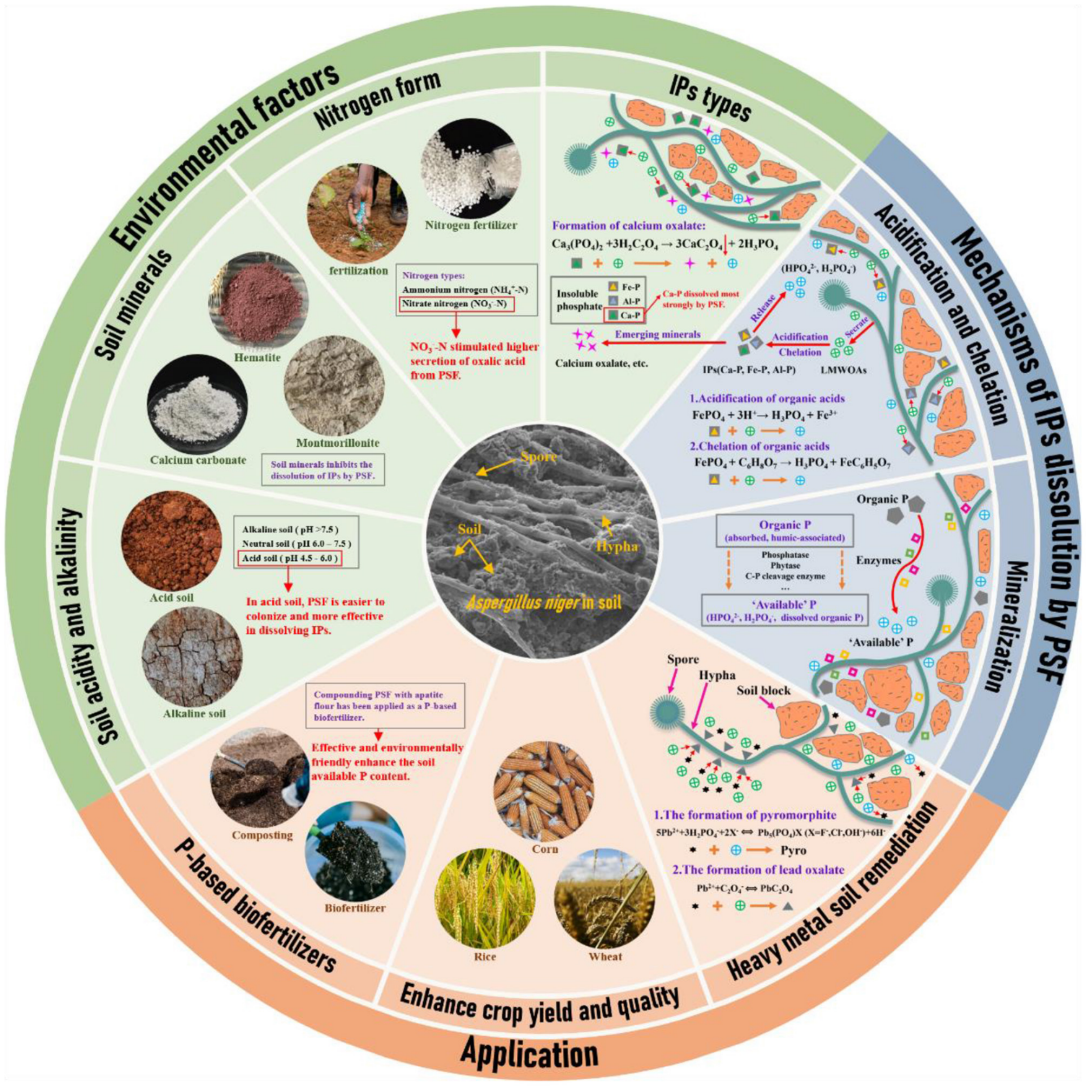
Mechanisms of phosphate-solubilizing fungi in insoluble phosphate dissolution and effects pathway in application.

Phosphate-solubilizing microorganisms (PSMs) can convert IPs into plant-absorbable and utilizable P forms in soil (Gadd et al., 2014; Owen et al., 2015; Jiang et al., 2021). Using PSMs in agricultural systems is an efficient and sustainable pathway to improve plant uptake of P from soil (Sharma et al., 2013; Tian et al., 2020; Munar et al., 2023; Wu et al., 2025). The common PSMs include phosphate-solubilizing fungi (PSF), phosphate-solubilizing bacteria (PSB), and phosphate-solubilizing actinomycete (PSA). Phosphate-solubilizing fungi have greater P-dissolving capacity than bacteria and actinomycetes. In the case of PSF *Aspergillus niger*, the amount of P dissolved from Ca-P (770.5 mg/L) is approximately two times higher than the PSB *Acinetobacter spp* (Li et al., 2019). Therefore, PSF is generally considered the primary candidate for IP dissolution (Figure 1).

PSF can secrete large amounts of organic acids, producing formic acid, oxalic acid, etc., reaching up to three times that of PSB (Li et al., 2016a). Meanwhile, PSF hyphae can directly penetrate insoluble phosphate minerals through mechanical pressure (Gadd, 2021). The highly developed hyphal network of PSF can extend several meters, significantly surpassing the range of PSB colonies (typically < 1 mm; Martinez and Marschmann, 2025). In addition, PSF fungal hyphae can penetrate deep into soil aggregates, while PSB usually accumulates on pore surfaces (Tian et al., 2021b). More importantly, PSF shows significant advantages in IPs dissolution, maintaining over 90% of IPs-dissolving capacity even after 10 successive subcultures (Kucey, 1983). Meanwhile, PSF also demonstrates a higher tolerance to drought and extreme pH levels than PSB (Li et al., 2016a; Bi et al., 2022). However, PSB usually offers practical benefits in production, including faster reproduction (generation time: 30–60 min) and more excellent suitability for liquid inoculant formulation compared to PSF (Belen Lobo et al., 2019). The co-inoculation of PSB and PSF significantly enhances IPs dissolution and plant growth compared to using either microorganism alone in sterile soil (Nacoon et al., 2020). Therefore, the co-inoculation of PSF and PSB presents a more promising approach to enhance the application of PSF in IPs dissolution.

PSF includes various genera such as *Penicillium, Aspergillus, Mucor, Trichoderma, Rhizopus, Phytophthora, Fusarium*, and *Saccharomyces* (Mercl et al., 2020; Zhang et al., 2020; Yang et al., 2022; Wang et al., 2023b). This diversity allows the selection of PSF strains tailored to specific environmental conditions and cropping systems (Figure 1). However, environmental factors such as soil pH, soil minerals, types of nutrients, organic fertilizer, and toxic pollutants (such as pesticides, heavy metals, and microplastics) would directly or indirectly affect the dissolution of IPs by PSF in soil (Tian et al., 2022a; Su et al., 2023; Wang et al., 2024a,b; Feng et al., 2025; Ni et al., 2025). In the case of typical PSF *Aspergillus niger*, the biomass and physiological activity were significantly higher in acidic soil than in alkaline soil (Su et al., 2023). Therefore, investigating the factors that affect the efficiency of IPs dissolution by PSF in soil is crucial for optimizing their application.

## 2 The secretion of dicarboxylic and tricarboxylic acids dominates the dissolution of IPs by PSF

Organic acid secretion is the primary pathway of PSF in the dissolution of IPs (Palmieri et al., 2019). On the one hand, PSF continuously releases low-molecule-weight organic acids (LMWOAs) to acidify the soil environment and significantly promote IPs dissolution (Kpomblekou-A and Tabatabai, 1994; Tian et al., 2020). On the other hand, the active functional groups of organic acids can also effectively chelate with metal cations (Ca^2+^, Fe^3+^, Al^3+^, etc.), thereby promoting the release of P from Ca-P, Fe-P, and Al-P, etc. (Shen et al., 2002; Kishore et al., 2015). Moreover, the LMWOAs can also directly release orthophosphate from soil minerals and Fe/Al oxides via ligand exchange (Sharma et al., 2013; Li et al., 2021).

The LMWOAs secreted by PSF include monocarboxylic, dicarboxylic, and tricarboxylic acids (Scervino et al., 2010). The dicarboxylic and tricarboxylic acids have higher acidity constants and chelating ability for metal cations (Kpomblekou-A and Tabatabai, 1994; Patel et al., 2008). Thus, dicarboxylic acids (oxalic, malonic, fumaric, and tartaric) and tricarboxylic acids (cis-aconitic and citric) are more effective in P detoxification than monocarboxylic acids (glycolic, pyruvic, and salicylic acid) (Table 1). The tricarboxylic acid (TCA) cycle in mitochondria is the key pathway for organic acid secretion by PSF (Mäkelä et al., 2010). The activity of various enzymes in the TCA cycle determines the types and amounts of organic acids secreted by PSF. For example, Fe-P supply significantly increased the citrate synthase activity and promoted the secretion of citric acid by PSF (Tian et al., 2021a). Meanwhile, constructing *Aspergillus niger* strains with the oxaloacetate acetylhydrolase-encoding gene can increase oxalic acid production by up to 3.1 times (Xu et al., 2019). Therefore, modifying environmental factors and genetically engineering fungi to enhance organic acid production are crucial strategies for strengthening PSF efficiency in IPs dissolution.

**Table 1:** Capacity of phosphate-solubilizing fungi in organic acid secretion and P release.

**PSF strains**	**Primary organic acid types**	**Organic acid production**	**P release (mg/L)**	**References**
*Trichoderma harzianum*	Glucose, citric, lactic and succinic acids	4,422.54 (mg/L)	9.31 (from RP)	Promwee et al., 2014
*Trichoderma asperellum*	Oxalic and citric acids	36 (mmol/L)	3.1 (from RP)	García-López et al., 2015
*Aspergillus niger*	Oxalic and citric acids	561.6 (mg/L)	861 (from TCP)	Tian et al., 2021b
*Penicillium oxalicum*	Oxalic, acetic, and lactic acids	0.143 (mmol/L)	189.1 (from TCP)	Yang et al., 2022
*Penicillium sp. PSM11-5*	Gluconic and citric acids	13,830 (mg/L)	300.1 (from TCP)	Chai et al., 2011
*Gigaspora margarita*	Citric acid	2.7 (mmol/L)	1.49 (from Fe-P)	Tawaraya et al., 2006
*Penicillium chrysogenum*	Oxalic acid	227.7 (mg/L)	693.6 (from TCP)	Wang et al., 2023a

IPs, insoluble phosphates; RP, rock phosphate; TCP, tricalcium phosphate.

## 3 Environmental factors affecting IPs dissolution by PSF in soil

### 3.1 Soil acidity and alkalinity

PSF dissolves IPs are generally more efficient in acidic soil than in alkaline soil. In acidic soil, the fungal abundance, microbial respiration, organic acid secretion, and phytase activity of PSF are higher than in alkaline soil (Adnan et al., 2022; Jin et al., 2022; Chandra et al., 2024). In the case of PSF, the abundance of *Aspergillus niger* in acidic red soil (pH 4.58) is approximately ten times greater than in alkaline red soil (pH 8.28) (Su et al., 2023). Generally, low soil pH values favor fungal growth, and high soil pH values promote bacterial growth (Su et al., 2023).

### 3.2 Soil minerals

The influence of soil minerals on the biological process of IPs dissolution by PSF is both beneficial and detrimental (Su et al., 2021). The soil mineral montmorillonite can enhance respiratory metabolism and oxalic acid secretion of PSF to improve the P-release capacity (Su et al., 2021). In contrast, other minerals (such as Calcium-bearing minerals) can also inhibit IPs dissolution by PSF via the adsorption of organic acids (do Nascimento et al., 2021; He et al., 2022). Specifically, carbonate can deplete the secreted oxalic acid to form stable calcium oxalate crystals, limiting IPs dissolution by PSF (Tian et al., 2021b). In general, soil minerals have a negative impact on the dissolution of IPs by PSF.

### 3.3 ${\text{NO}}_{3}^{-}$-N and ${\text{NH}}_{4}^{+}$-N

Nitrogen can significantly affect the phosphate-solubilizing capacity of PSF. Typically, the supply of ${\text{NO}}_{3}^{-}$-N is more efficient than ${\text{NH}}_{4}^{+}$-N in IPs dissolution by PSF. Nitrogen forms can significantly affect the secretion of organic acids by PSF. For example, PSF *Aspergillus niger* predominantly secrete citric and malic acids in the supply of ${\text{NH}}_{4}^{+}$-N, while it primarily secrete oxalic acid under ${\text{NO}}_{3}^{-}$-N conditions (Gadd et al., 2014). ${\text{NO}}_{3}^{-}$-N stimulates the secretion of oxalic acid by *Aspergillus niger* primarily through the upregulation of the oxaloacetate acetylhydrolase (OAH) gene (Kobayashi et al., 2014). Oxalic acid is the primary organic acid that functions in IPs dissolution by PSF due to the high acidity constant (Feng et al., 2022). Consequently, the form of nitrogen can modulate the activity of enzymes involved in the TCA cycle of PSF, thereby impacting their IPs dissolution capacity.

### 3.4 IPs types

The different phosphates can affect the types and amounts of organic acids secreted by PSF (Tian et al., 2021a). Compared with Fe-P and Al-P, PSF is more effective in promoting P release from Ca-P (Tian et al., 2021a). On the one hand, Ca-P promotes PSF to secrete more oxalic acid compared with Fe-P (Wang et al., 2023a). On the other hand, oxalic acid secreted by PSF can combine with Ca^2+^ to form relatively stable calcium oxalate crystals, which can promote the release of P from Ca-P (Tian et al., 2021a; Wang et al., 2022). Therefore, Ca-based phosphate fertilizer shows excellent potential in producing “ phosphate-based biofertilizer ”.

## 4 Application of PSF in soil P cycle, crop yield, and heavy metal remediation

PSF can significantly increase soil P effectiveness and plant P uptake (Ahmad et al., 2013; Fiuza et al., 2022). For instance, *Trichoderma harzianum* inoculation can increase wheat biomass and plant P content and improve crop yield (Akbar et al., 2023). The combination of native arbuscular mycorrhizal fungi (AMF) and PSF (*Aspergillus niger* and *Penicillium brevis*) can significantly enhance soil available P, stimulate phosphatase activity in the coffee rhizosphere and promote coffee growth (Rojas et al., 2019). More importantly, multiple field experiments demonstrated that the application of “ phosphate-based biofertilizer ” (PSF and apatite) can significantly improve soil P utilization and enhance crop quality and yield (da Silva et al., 2017; Arias et al., 2023; Wang et al., 2023b). Compared with chemical phosphate fertilizers, inoculating with PSF can increase approximately 30% P uptake efficiency and approximately 16% yield of eggplant (Yin et al., 2021). Meanwhile, the absorption efficiency of soybeans for phosphate rock powder also improved by 56.1% after *Trichoderma* inoculation (Bononi et al., 2020). Even in barren desert soils, adding silicon (Si) can enhance the phosphate solubilization of fungi by 50%, providing a promising solution to P deficiency in desert soils (Ameen et al., 2019).

The combination of PSF and apatite also shows excellent potential in the remediation of heavy metal-contaminated soil (Tian et al., 2018, 2022b). Oxalic acid secreted by PSF can also react with heavy metal cations (e.g., Pb^2+^) to form insoluble oxalate minerals, e.g., lead oxalate (Li et al., 2016b; Tian et al., 2023). The combination of *Penicillium oxalicum* and tricalcium phosphate not only increases soil available P but also reduces the environmental exposure toxicity of soil Pb (Hao et al., 2022).

## 5 Potential pathways to enhance the insoluble phosphate solubilization and application of PSF

PSFs can secrete large amounts of organic acids to promote P release from IPs in soil. The critical enhancements that improve the dissolution of IPs by PSF are fungal bioactivity and organic acid secretion capacity. However, the application of PSF in agricultural production also faces several limitations, including inconsistent performance and poor environmental adaptability. Hence, improving the IPs dissolution by PSF remains a considerable challenge in the future. Firstly, screening and cultivating more efficient and adaptable PSF and using genetic engineering to improve existing strains is necessary. Secondly, modifying environmental factors like soil pH and organic matter content to create more favorable conditions for the PSF. Thirdly, developing multifunctional composite biofertilizer products of PSF. Lastly, conducting long-term field experiments to accumulate application data of PSF under different soil types and climatic conditions. Overall, improving the practical application effect of PSF in agricultural production requires further attention in the future.

## References

[B1] Adnan M. Fahad S. Saleem M. H. Ali B. Mussart M. Ullah R.. (2022). Comparative efficacy of phosphorous supplements with phosphate solubilizing bacteria for optimizing wheat yield in calcareous soils. Sci. Rep. 12:11997. 10.1038/s41598-022-16035-3 35835850 PMC9283399

[B2] Ahmad E. Khan M. S. Zaidi A. (2013). ACC deaminase producing *Pseudomonas putida* strain PSE3 and *Rhizobium leguminosarum* strain RP2 in synergism improves growth, nodulation and yield of pea grown in alluvial soils. Symbiosis 61, 93–104. 10.1007/s13199-013-0259-6

[B3] Akbar M. Chohan S. A. Yasin N. A. Ahmad A. Akram W. Nazir A.. (2023). Mycorrhizal inoculation enhanced tillering in field grown wheat, nutrition enrichment and soil properties. Peerj 11:e15686. 10.7717/peerj.15686 37719109 PMC10504892

[B4] Ameen F. AlYahya S. A. AlNadhari S. Alasmari H. Alhoshani F. Wainwright M. (2019). Phosphate solubilizing bacteria and fungi in desert soils: species, limitations and mechanisms. Arch. Agron. Soil Sci. 65, 1446–1459. 10.1080/03650340.2019.1566713

[B5] Arias R. M. Abarca G. H. Rojas Y. D. P. Elizondo Y. D. Guzman K. Y. G. (2023). Selection and characterization of phosphate-solubilizing fungi and their effects on coffee plantations. Plants-Basel 12:3395. 10.3390/plants12193395 37836135 PMC10574286

[B6] Belen Lobo C. Juarez Tomas M. S. Viruel E. Alejandra Ferrero M. Ester Lucca M. (2019). Development of low-cost formulations of plant growth-promoting bacteria to be used as inoculants in beneficial agricultural technologies. Microbiol. Res. 219, 12–25. 10.1016/j.micres.2018.10.012 30642462

[B7] Bi W. Weng B. Yan D. Wang H. Wang M. Yan S.. (2022). Responses of phosphate-solubilizing microorganisms mediated phosphorus cycling to drought-flood abrupt alternation in summer maize field soil. Front. Microbiol. 12:768921. 10.3389/fmicb.2021.768921 35111138 PMC8802831

[B8] Bisson C. Adams N. B. P. Stevenson B. Brindley A. A. Polyviou D. Bibby T. S.. (2017). The molecular basis of phosphite and hypophosphite recognition by ABC-transporters. Nat. Commun. 8:1746. 10.1038/s41467-017-01226-8 29170493 PMC5700983

[B9] Bononi L. Chiaramonte J. B. Pansa C. C. Moitinho M. A. Melo I. S. (2020). Phosphorus-solubilizing Trichoderma spp. from Amazon soils improve soybean plant growth. Sci. Rep. 10:2858. 10.1038/s41598-020-59793-8 32071331 PMC7028723

[B10] Chai B. Wu Y. Liu P. M. Liu B. A. Gao M. Y. (2011). Isolation and phosphate-solubilizing ability of a fungus, Penicillium sp from soil of an alum mine. J. Basic Microbiol. 51, 5–14. 10.1002/jobm.201000192 21259286

[B11] Chandra P. Rai A. K. Basak N. Sundha P. Prajapat K. Singh A.. (2024). *In vitro* P- solubilization activity of halophilic fungi in salt- affected soils and their potential as bio-inoculants. Environ. Eng. Res. 29:230760. 10.4491/eer.2023.760

[B12] da Silva V. N. de Souza Fernandes da Silva L. E. da Silva A. J. N. Stamford N. P. de Macedo G. R. (2017). Solubility curve of rock powder inoculated with microorganisms in the production of biofertilizers. Agric. Nat. Resour. 51, 142–147. 10.1016/j.anres.2017.01.001

[B13] do Nascimento J. M. Ferreira Vieira Netto J. A. Valadares R. V. Mendes G. d. O. da Silva I. R. Vergutz L.. (2021). *Aspergillus niger* as a key to unlock fixed phosphorus in highly weathered soils. Soil Biol. Biochem. 156:108190. 10.1016/j.soilbio.2021.108190

[B14] Feng B. X. Xue Y. Wang D. C. Chen S. H. Zhang S. Zhang L. L.. (2025). Stability of lead immobilization by *Aspergillus niger* and fluorapatite under different pH conditions. Ecotoxicol. Environ. Saf. 289:117706. 10.1016/j.ecoenv.2025.117706 39799925

[B15] Feng Y. Zhang L. Li X. Wang L. Yusef K. K. Gao H.. (2022). Remediation of lead contamination by *Aspergillus niger* and phosphate rocks under different nitrogen sources. Agronomy 12:1639. 10.3390/agronomy12071639

[B16] Fiuza D. A. F. Vitorino L. C. Souchie E. L. Neto M. R. Bessa L. A. Silva C. F. D.. (2022). Effect of rhizobacteria inoculation via soil and seeds on glycine max L. plants grown on soils with different cropping history. Microorganisms 10:691. 10.3390/microorganisms10040691 35456743 PMC9031610

[B17] Gadd G. M. (2021). Fungal biomineralization. Curr. Biol. 31, R1557–R1563. 10.1016/j.cub.2021.10.041 34932960

[B18] Gadd G. M. Bahri-Esfahani J. Li Q. Rhee Y. J. Wei Z. Fomina M.. (2014). Oxalate production by fungi: significance in geomycology, biodeterioration and bioremediation. Fungal Biol. Rev. 28, 36–55. 10.1016/j.fbr.2014.05.001

[B19] García-López A. M. Avilés M. Delgado A. (2015). Plant uptake of phosphorus from sparingly available P- sources as affected by *Trichoderma asperellum* T34. Agr. Food Sci. 24, 249–260. 10.23986/afsci.49532

[B20] Hao S. F. Wang P. Y. Ge F. Li F. Deng S. Q. Zhang D. Y.. (2022). Enhanced Lead (Pb) immobilization in red soil by phosphate solubilizing fungi associated with tricalcium phosphate influencing microbial community composition and Pb translocation in *Lactuca sativa* L. J. Hazard. Mater. 424:127720. 10.1016/j.jhazmat.2021.127720 34810010

[B21] He N. Hu L. Jiang C. Li M. (2022). Remediation of chromium, zinc, arsenic, lead and antimony contaminated acidic mine soil based on *Phanerochaete chrysosporium* induced phosphate precipitation. Sci. Total Environ. 850:157995. 10.1016/j.scitotenv.2022.157995 35964759

[B22] Jayashree S. Vadivukkarasi P. Anand K. Kato Y. Seshadri S. (2011). Evaluation of pink-pigmented facultative methylotrophic bacteria for phosphate solubilization. Arch. Microbiol. 193, 543–552. 10.1007/s00203-011-0691-z 21445558

[B23] Jiang F. Y. Zhang L. Zhou J. C. George T. S. Feng G. (2021). Arbuscular mycorrhizal fungi enhance mineralisation of organic phosphorus by carrying bacteria along their extraradical hyphae. New Phytol. 230, 304–315. 10.1111/nph.17081 33205416

[B24] Jin F. Hu Q. Zhao Y. Lin X. Zhang J. Zhang J.. (2022). Enhancing quinoa growth under severe saline-alkali stress by phosphate solubilizing microorganism *Penicillium funicuiosum* P1. PLoS ONE 17:e0273459. 10.1371/journal.pone.0273459 36067185 PMC9447905

[B25] Kishore N. Pindi P. K. Ram Reddy S. (2015). “Phosphate-solubilizing microorganisms: a critical review,” in Plant Biology and Biotechnology: Volume I: Plant Diversity, Organization, Function and Improvement, eds. B. Bahadur, M. Venkat Rajam, L. Sahijram, and K. V. Krishnamurthy (New Delhi: Springer India), 307–333. 10.1007/978-81-322-2286-6_12

[B26] Kobayashi K. Hattori T. Honda Y. Kirimura K. (2014). Oxalic acid production by citric acid-producing *Aspergillus niger* overexpressing the oxaloacetate hydrolase gene oahA. J. Ind. Microbiol. Biotechnol. 41, 749–756. 10.1007/s10295-014-1419-2 24615146

[B27] Kpomblekou-A K. Tabatabai M. A. (1994). Effect of organic acids on release of phosphorus from phosphate rocks. Soil Sci. 158, 442–453. 10.1097/00010694-199415860-00006

[B28] Kucey R. M. N. (1983). Phosphate-solubilizing bacteria and fungi in various cultivated and virgin alberta soils. Can. J. Soil Sci. 63, 671–678. 10.4141/cjss83-068

[B29] Li C. Li Q. Wang Z. Ji G. Zhao H. Gao F.. (2019). Environmental fungi and bacteria facilitate lecithin decomposition and the transformation of phosphorus to apatite. Sci. Rep. 9:15291. 10.1038/s41598-019-51804-7 31653926 PMC6814757

[B30] Li H. Bölscher T. Winnick M. Tfaily M. M. Cardon Z. G. Keiluweit M.. (2021). Simple plant and microbial exudates destabilize mineral-associated organic matter via multiple pathways. Environ. Sci. Technol. 55, 12131–12131. 10.1021/acs.est.1c05166 34383465

[B31] Li Z. Bai T. Dai L. Wang F. Tao J. Meng S.. (2016a). A study of organic acid production in contrasts between two phosphate solubilizing fungi: *Penicillium oxalicum* and *Aspergillus niger*. Sci. Rep. 6:25313. 10.1038/srep25313 27126606 PMC4850453

[B32] Li Z. Wang F. Bai T. Tao J. Guo J. Yang M.. (2016b). Lead immobilization by geological fluorapatite and fungus *Aspergillus niger*. J. Hazard. Mater. 320, 386–392. 10.1016/j.jhazmat.2016.08.051 27585270

[B33] Mahdi I. Fahsi N. Hafidi M. Allaoui A. Biskri L. (2020). Plant growth enhancement using rhizospheric halotolerant phosphate solubilizing bacterium *Bacillus licheniformis* QA1 and *Enterobacter asburiae* QF11 isolated from chenopodium quinoa willd. Microorganisms 8:948. 10.3390/microorganisms8060948 32599701 PMC7356859

[B34] Mäkelä M. R. Hildén K. Lundell T. K. (2010). Oxalate decarboxylase: biotechnological update and prevalence of the enzyme in filamentous fungi. Appl. Microbiol. Biotechnol. 87, 801–814. 10.1007/s00253-010-2650-z 20464388

[B35] Martinez N. D. Marschmann G. L. (2025). How fungi build planet-altering ‘road' networks. Nature 639, 39–40. 10.1038/d41586-025-00307-9 40011650

[B36] Mercl F. García-Sánchez M. Kulhánek M. Kosnár Z. Száková J. Tlustos P.. (2020). Improved phosphorus fertilisation efficiency of wood ash by fungal strains *Penicillium* sp. PK112 and *Trichoderma harzianum* OMG08 on acidic soil. Appl. Soil Ecol. 147:103360. 10.1016/j.apsoil.2019.09.010

[B37] Munar A. Sembiring M. Tantawi A. R. Sabrina T. (2023). Isolation and identification of phosphate solubilizing microbes in the rhizosphere of maize by sound exposure. Emir. J. Food Agric. 35, 964–970. 10.9755/ejfa.2023.3169

[B38] Nacoon S. Jogloy S. Riddech N. Mongkolthanaruk W. Kuyper T. W. Boonlue S.. (2020). Interaction between phosphate solubilizing bacteria and arbuscular mycorrhizal fungi on growth promotion and tuber inulin content of *Helianthus tuberosus* L. Sci. Rep. 10:4916. 10.1038/s41598-020-61846-x 32188930 PMC7080738

[B39] Ni B. Xiao L. Lin D. Zhang T. L. Zhang Q. Liu Y. J.. (2025). Increasing pesticide diversity impairs soil microbial functions. Proc. Natl. Acad. Sci. U. S. A. 122:e2419917122. 10.1073/pnas.2419917122 39786931 PMC11745395

[B40] Owen D. Williams A. P. Griffith G. W. Withers P. J. A. (2015). Use of commercial bio-inoculants to increase agricultural production through improved phosphrous acquisition. Appl. Soil Ecol. 86, 41–54. 10.1016/j.apsoil.2014.09.012

[B41] Palmieri F. Estoppey A. House G. L. Lohberger A. Bindschedler S. Chain P. S. G.. (2019). “Chapter two - oxalic acid, a molecule at the crossroads of bacterial-fungal interactions,” in Advances in Applied Microbiology, eds. G. M. Gadd and S. Sariaslani (Academic Press), 49–77. 10.1016/bs.aambs.2018.10.001 30798804

[B42] Patel D. K. Archana G. Kumar G. N. (2008). Variation in the nature of organic acid secretion and mineral phosphate solubilization by citrobacter sp. DHRSS in the presence of different sugars. Curr. Microbiol. 56, 168–174. 10.1007/s00284-007-9053-0 17965911

[B43] Promwee A. Issarakraisila M. Intana W. Chamswarng C. Yenjit P. (2014). Phosphate solubilization and growth promotion of rubber tree (*Hevea brasiliensis* Muell. Arg.) by trichoderma strains. J. Agric. Sci. 6:8. 10.5539/jas.v6n9p8

[B44] Rojas Y. D. P. Arias R. M. Ortiz R. M. Aguilar D. T. Heredia G. Yon Y. R.. (2019). Effects of native arbuscular mycorrhizal and phosphate-solubilizing fungi on coffee plants. Agrofor. Syst. 93, 961–972. 10.1007/s10457-018-0190-1

[B45] Scervino J. M. Mesa M. P. Della Mónica I. Recchi M. Sarmiento Moreno N. Godeas A.. (2010). Soil fungal isolates produce different organic acid patterns involved in phosphate salts solubilization. Biol. Fertil. Soils 46, 755–763. 10.1007/s00374-010-0482-8

[B46] Sharma S. B. Sayyed R. Z. Trivedi M. H. Gobi T. A. (2013). Phosphate solubilizing microbes: sustainable approach for managing phosphorus deficiency in agricultural soils. Springerplus 2:587. 10.1186/2193-1801-2-587 25674415 PMC4320215

[B47] Shen H. Yan X. Zhao M. Zheng S. Wang X. (2002). Exudation of organic acids in common bean as related to mobilization of aluminum- and iron-bound phosphates. Environ. Exp. Bot. 48, 1–9. 10.1016/S0098-8472(02)00009-6

[B48] Su M. Mei J. Mendes G.d.O. Tian D. Zhou L. Hu S.. (2023). Alkalinity exacerbates phosphorus deficiency in subtropical red soils: insights from phosphate-solubilizing fungi. Soil Use Manag. 39, 1504–1516. 10.1111/sum.12911

[B49] Su M. Meng L. Zhao L. Tang Y. Qiu J. Tian D.. (2021). Phosphorus deficiency in soils with red color: insights from the interactions between minerals and microorganisms. Geoderma 404:115311. 10.1016/j.geoderma.2021.115311

[B50] Tawaraya K. Naito M. Wagatsuma T. (2006). Solubilization of insoluble inorganic phosphate by hyphal exudates of arbuscular mycorrhizal fungi. J. Plant Nutr. 29, 657–665. 10.1080/01904160600564428

[B51] Tian D. Cheng X. Wang L. Hu J. Zhou N. Xia J.. (2022a). Remediation of lead-contaminated water by red yeast and different types of phosphate. Front. Bioeng. Biotechnol. 10:775058. 10.3389/fbioe.2022.775058 35387302 PMC8979109

[B52] Tian D. Gao H. Zhang C. Ye X. (2024). “Chapter 21 - Sustainable release of phosphorus under heavy metal stresses: from microbiology to productivity,” in Beneficial Microbes for Sustainable Agriculture Under Stress Conditions, ed. T. Sa (Academic Press), 427–443. 10.1016/B978-0-443-13193-6.00021-X

[B53] Tian D. Li Z. O'Connor D. Shen Z. Hou D. (2020). The need to prioritize sustainable phosphate-based fertilizers. Soil Use Manag. 36, 351–354. 10.1111/sum.12578

[B54] Tian D. Su M. Zou X. Zhang L. Tang L. Geng Y.. (2021b). Influences of phosphate addition on fungal weathering of carbonate in the red soil from karst region. Sci. Total Environ. 755:142570. 10.1016/j.scitotenv.2020.142570 33035850

[B55] Tian D. Wang L. Hu J. Zhang L. Zhou N. Xia J.. (2021a). A study of P release from Fe-P and Ca-P via the organic acids secreted by *Aspergillus niger*. J. Microbiol. 59, 819–826. 10.1007/s12275-021-1178-5 34382148

[B56] Tian D. Wang W. Su M. Zheng J. Wu Y. Wang S.. (2018). Remediation of lead-contaminated water by geological fluorapatite and fungus *Penicillium oxalicum*. Environ. Sci. Pollut. Res. 25, 21118–21126. 10.1007/s11356-018-2243-4 29770937

[B57] Tian D. Xia J. Zhou N. Xu M. Li X. Zhang L.. (2022b). The utilization of phosphogypsum as a sustainable phosphate-based fertilizer by *Aspergillus niger*. Agronomy 12:646. 10.3390/agronomy12030646

[B58] Tian D. Zhang X. Wang L. Han M. Zhang C. Ye X.. (2023). Lead remediation is promoted by phosphate-solubilizing fungi and apatite via the enhanced production of organic acid. Front. Bioeng. Biotechnol. 11:1180431. 10.3389/fbioe.2023.1180431 37064227 PMC10097878

[B59] Wang L. Guan H. Hu J. Feng Y. Li X. Yusef K. K.. (2022). *Aspergillus niger* enhances organic and inorganic phosphorus release from wheat straw by secretion of degrading enzymes and oxalic acid. J. Agric. Food Chem. 70, 10738–10746. 10.1021/acs.jafc.2c03063 36027054

[B60] Wang L. Han M. Zhang X. Hu J. Zhang S. Wang D.. (2023a). Mechanism and dissolve capacity of *Penicillium chrysogenum* to different insoluble phosphates. J. Plant Nutr. Fertilizers 29, 1343–1351. 10.11674/zwyf.202257537665553

[B61] Wang X. L. Qiu S. Y. Zhou S. Q. Xu Z. H. Liu X. T. (2023b). Phosphate-solubilizing capacity of *Paecilomyces lilacinus* PSF7 and optimization using response surface methodology. Microorganisms 11:454. 10.3390/microorganisms11020454 36838419 PMC9962588

[B62] Wang Y. F. Liu Y. J. Fu Y. M. Xu J. Y. Zhang T. L. Cui H. L.. (2024a). Microplastic diversity increases the abundance of antibiotic resistance genes in soil. Nat. Commun. 15:9788. 10.1038/s41467-024-54237-7 39532872 PMC11557862

[B63] Wang Y. F. Xu J. Y. Liu Z. L. Cui H. L. Chen P. Cai T. G.. (2024b). Biological interactions mediate soil functions by altering rare microbial communities. Environ. Sci. Technol. 58, 5866–5877. 10.1021/acs.est.4c00375 38504110

[B64] Wu S. M. Zhang W. Wang D. R. Balcazar J. L. Wang G. H. Ye M.. (2025). Bacteriophage-bacteria interactions promote ecological multifunctionality in compost-applied soils. Environ. Microbiol. 27:e70074. 10.1111/1462-2920.70074 40109201

[B65] Xu Y. X. Shan L. Zhou Y. T. Xie Z. J. Ball N. S. Cao W.. (2019). Development of a Cre-lox P-based genetic system in *Aspergillus niger* ATCC1015 and its application to construction of efficient organic acid-producing cell factories. Appl. Microbiol. Biotechnol. 103, 8105–8114. 10.1007/s00253-019-10054-3 31392377

[B66] Yang T. Y. Li L. B. Wang B. S. Tian J. Shi F. H. Zhang S. S.. (2022). Isolation, mutagenesis, and organic acid secretion of a highly efficient phosphate-solubilizing fungus. Front. Microbiol. 13:793122. 10.3389/fmicb.2022.793122 35547144 PMC9082945

[B67] Yin J. Sui Z. Huang J. (2021). Mobilization of soil inorganic phosphorus and stimulation of crop phosphorus uptake and growth induced by *Ceriporia lacerata* HG2011. Geoderma 383:114690. 10.1016/j.geoderma.2020.114690

[B68] Zhang J. E. Feng L. F. Ouyang Y. Hu R. R. Xu H. Q. Wang J. X.. (2020). Phosphate-solubilizing bacteria and fungi in relation to phosphorus availability under different land uses for some latosols from Guangdong, China. Catena 195:104686. 10.1016/j.catena.2020.104686

[B69] Zhu J. Li M. Whelan M. (2018). Phosphorus activators contribute to legacy phosphorus availability in agricultural soils: a review. Sci. Total Environ. 612, 522–537. 10.1016/j.scitotenv.2017.08.095 28865270

